# Antagonistic Mobile Genetic Elements Can Counteract Each Other’s Effects on Microbial Community Composition

**DOI:** 10.1128/mbio.00460-23

**Published:** 2023-04-06

**Authors:** Meaghan Castledine, Arthur Newbury, Rai Lewis, Christian Hacker, Sean Meaden, Angus Buckling

**Affiliations:** a College of Life and Environmental Sciences, Environment and Sustainability Institute, University of Exeter, Penryn, United Kingdom; b Bioimaging Center, College of Life and Environmental Sciences, University of Exeter, Exeter, United Kingdom; Corporación CorpoGen

**Keywords:** bacteriophages, indirect interactions, “kill the winner” dynamics, microbial communities, mobile genetic elements, plasmids

## Abstract

Bacteriophages (“phages”) are hypothesized to be key drivers of bacterial population dynamics, driving microbial community composition, but empirical support for this is mixed. One reason why phages may have a less-than-expected impact on community composition is that many different phages and other mobile genetic elements (MGEs) interact with each bacterium. For instance, the same phage may have higher or lower costs to different bacterial strains or species. Assuming that resistance or susceptibility to MGE infection is not consistent across all MGEs, a simple prediction is that the net effect of MGEs on each bacterial taxon may converge with an increasing number of interactions with different MGEs. We formalized this prediction using *in silico* population dynamics simulations and then carried out experiments using three bacterial species, one generalist conjugative plasmid, and three species-specific phages. While the presence of only phages or only the plasmid altered community structure, these differential effects on community structure canceled out when both were together. The effects of MGEs were largely indirect and could not be explained by simple pairwise bipartite interactions (i.e., between each MGE and each bacterial species). Our results suggest that the effects of MGEs may be overestimated by studies that focus on a single MGE and not on interactions among multiple MGEs.

## INTRODUCTION

Bacteriophages are ubiquitous, abundant, often taxon-specific, and can have major impacts on microbial densities (1). Consequently, phages are considered to be key drivers of microbial community composition and function (1–3). Both theory and empirical work suggest that phages (or antagonists in general) can promote microbial diversity (both within and between species) through “kill the winner” dynamics (or negative frequency-dependent selection, under evolutionary scenarios) (1, 4, 5). Phages may also reduce microbial diversity if, for example, the dominant taxa are less susceptible to infection (6, 7) or phages reduce intraspecific competition, therefore increasing densities (8, 9).

Empirical evidence for a major role of phages in structuring microbial community composition outside laboratory settings is mixed (10). Some phage-addition experiments using natural communities have found that phages have limited effects on bacterial populations and/or diversity (11–14). Correlations between bacteria and phage dynamics have been reported, but it is unclear whether phages are driving these dynamics or simply tracking fluctuations in host densities (1, 10, 15–18). Situations in which viruses unambiguously drive microbial host population dynamics are typically short-lived and arise when bacteria and/or phage densities are unusually high (1, 19, 20); for example, following seasonally driven algal blooms (2, 20) or phage therapy (21).

Phages may impose limited top-down control on microbial dynamics for several reasons. Phages may simply not encounter many susceptible hosts, particularly in spatially structured environments (22). There may be few infective particles for a given taxon, either because of non-host resistance (i.e., phage taxon- and sometimes strain-level specificity) (23–26) or evolved resistance (3). While bacteria and phage can undergo coevolution, bacteria can rapidly evolve resistance to phage, which is hard for phage to overcome (3, 27). Furthermore, where coevolution is ongoing, it is likely characterized by fluctuations in genotype frequencies (potentially having little impact on the population size of a given taxon) (28–31).

Another consideration is that a given bacterial taxon can potentially be infected by many different mobile genetic elements (MGEs), including multiple types of phages and plasmids (32–35). MGEs can antagonistically interact, thereby limiting their individual effects (34, 36), but interactions between MGEs can also be positive or neutral and are contingent on both the specific MGEs and the environmental context (35, 37–39). Thus, multiple MGEs could increase, decrease, or have no impact on phage-mediated changes in community composition. However, one general prediction is that different MGEs will have differential effects on different taxa (40–43); hence, the net effect of MGEs on different microbial taxa may converge with increasing types of MGEs. Consequently, even if MGEs do have an important impact on total microbial biomass, they will potentially have a less important role in shaping composition. Here, we formalized this prediction with *in silico* simulations and then tested it using a synthetic community involving three bacterial taxa, three specialized lytic phages, and a generalist (parasitic) plasmid. Specifically, we compared community composition in the absence or presence of phages, plasmids, or both. We focus on antagonistic rather than mutualistic interactions between MGEs and hosts because antagonistic interactions are typically the focus of MGE-mediated control of microbial community composition.

## RESULTS

### Simulation.

**FIG 1:**
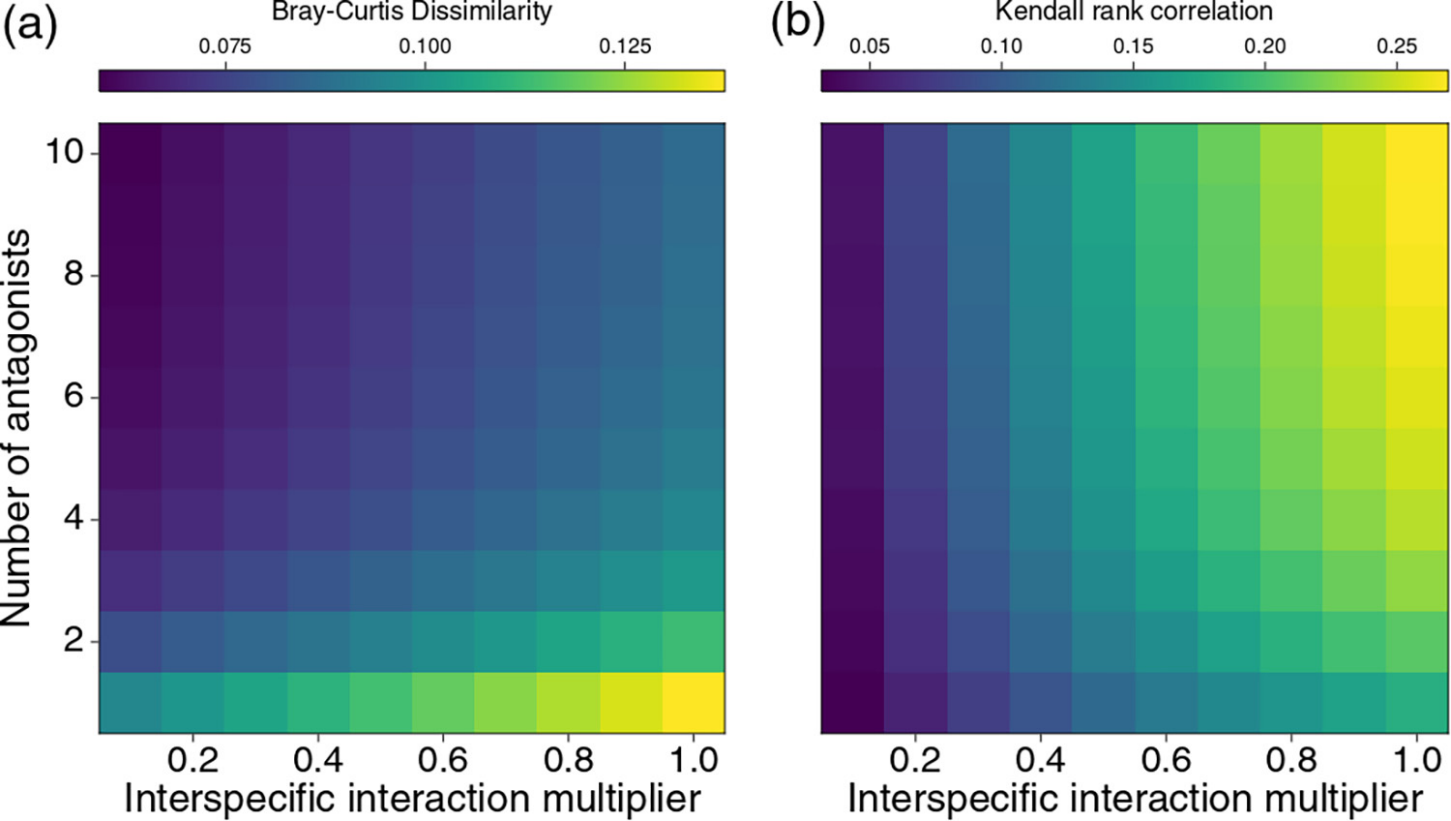
Impacts of antagonists on the structure of simulated communities, measured as (a) Bray-Curtis dissimilarity and (b) Kendall rank correlation between a community with 0 antagonists and one with *m* (*y* axis) antagonists, for a range of typical interspecific (focal-focal) interaction coefficients (*x* axis). Each tile represents the mean value from 100,000 simulations. Results are shown for *n *= 10 focal species and antagonist effects on focal species are drawn from N^+^ (0, σ = 0.3).

To assess the impact of the number of antagonists (*m*) on community structure, we measured both the Bray-Curtis dissimilarity and the Kendall rank correlation between the relative abundances of focal species with and without antagonists, for 100,000,000 simulated communities (see Materials and Methods). The results were qualitatively the same for all sets of parameters (Fig. 1 and Fig. S1 in the supplemental material), with higher values of *m* producing smaller changes in community structure (smaller Bray-Curtis dissimilarity and higher Kendall rank correlation). Thus, the main finding from the simulations is that increasing the number of antagonists typically reduces the net effect of antagonists on microbial community structure. Increasing the strength of interspecific interactions increases both the dissimilarity and the rank correlation, i.e., the relative densities of focal species are more affected by antagonists when there is more interspecific competition, but their rankings (most to least abundant) are less affected.

### Bacteriophage characterization.

**FIG 2:**
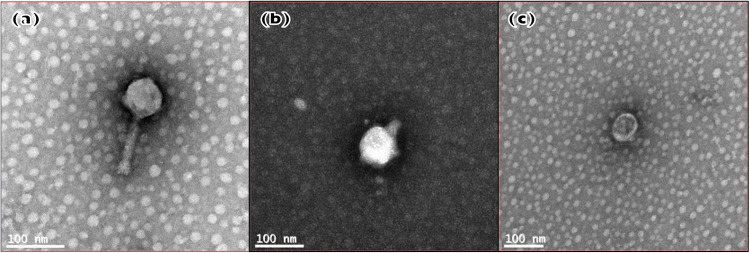
Transmission electron micrographs of phages (a) ORM_20, (b) CHF7MC, and (c) VAC_51. Scale bar =100 nm.

Assembled genomes were run through the BLAST database for sequence comparison. *Variovorax* and *Ochrobactrum* phages had <30% and <7% similarity, respectively, to known bacteriophages; as such, they were given unique identifiers as VAC_51 (*Variovorax* sp. phage) and ORM_20 (*Ochrobactrum* sp. phage). The Pseudomonas phage had a 97% similarity to Pseudomonas syringae phage CHF7, which has been characterized previously (44), and was named CHF7MC (CHF7-like Pseudomonas phage). Genes with known functions are listed in Table S1. CHF7MC has 16 hypothetical proteins, ORM_20 has 175, and VAC_51 has 33. No genes associated with lysogeny were identified. ORM_20 was identified as belonging to *Myoviridae* while VAC_51 and CHF7MC belonged to *Podoviridae* (Fig. 2).

10.1128/mbio.00460-23.3 TABLE S1 Genes of known function for Pseudomonas phage CHF7MC, *Ochrobactrum* phage ORM_20, and *Variovorax* phage VAC_51. Length = base pairs (bp). Download Table S1, DOCX file, 0.02 MB. Copyright © 2023 Castledine et al. 2023 Castledine et al. https://creativecommons.org/licenses/by/4.0/ This content is distributed under the terms of the Creative Commons Attribution 4.0 International license.

### Interaction between phage and plasmids.

**FIG 3:**
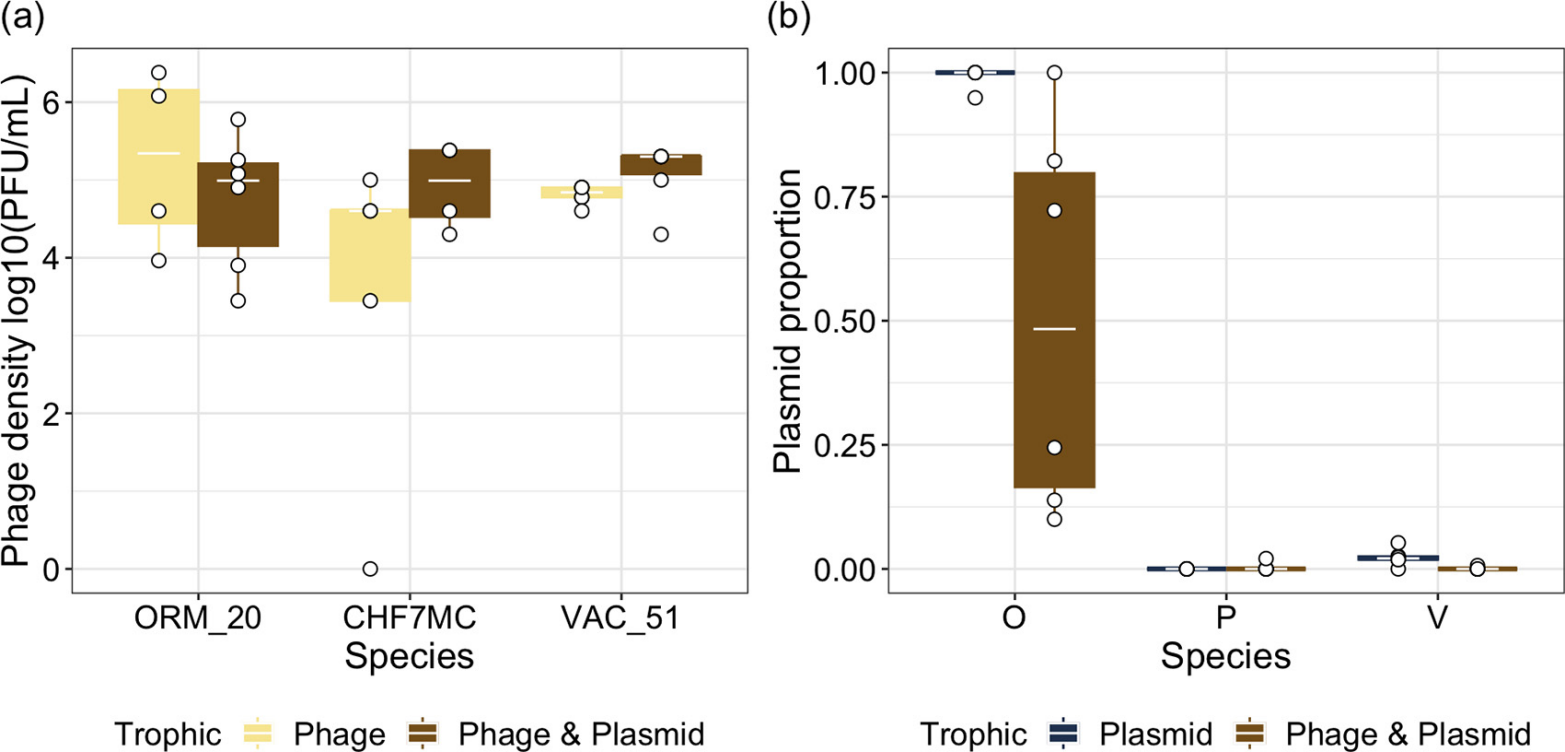
Within each polyculture, (a) phage densities and (b) plasmid proportions were tracked for each species at the end of 2 weeks of culturing. Points represent individual treatment replicates. Tops and bottoms of the bars represent the 75th and 25th percentiles of the data, white lines indicate medians, and whiskers extend from their respective hinge to the smallest or largest value no further than 1.5× the interquartile range. ORM_20, *Ochrobactrum* sp. phage; CHF7MC Pseudomonas sp. phage; VAC_51, *Variovorax* sp. phage. O, *Ochrobactrum* sp.; P, Pseudomonas sp.; V, *Variovorax* sp.

First, we considered the effects of the plasmid on phages, and vice versa, by measuring phage densities and plasmid proportions within the communities. Phage persisted in all replicates for all species and with plasmids present or absent (Fig. 3a). In contrast, the plasmid was lost in all replicates for Pseudomonas in plasmid-only communities and in 5/6 replicates of phage-plasmid communities, suggesting high plasmid instability regardless of phage presence (Fig. 3b). Similarly, the plasmid was lost for *Variovorax* in 1/6 replicates of the plasmid-only communities and 5/6 replicates of the phage-plasmid communities, suggesting that the phage accelerated plasmid loss; however, where present, plasmid densities were still low on average ($\bar{x}$ = 0.001, 95% confidence interval [CI] = 0.0004 to 0.005; Fig. 3b). *Ochrobactrum* was the only species to retain the plasmid when phages were both present and absent (Fig. 3b). Phage densities were not significantly different between species (analysis of variance [ANOVA] comparing models with and without species × plasmid interaction: $x_{2}^{2}\;$= 4.52, *P* = 0.105; fixed effect of species identity: $x_{2}^{2}\;$= 4.15, *P* = 0.126) or significantly affected by plasmid presence (ANOVA comparing models with and without plasmid: $x_{1}^{2}\;$= 0.827, *P* = 0.363; Fig. 3a). The presence of the plasmid did not significantly affect the evolution of phage resistance in two species because no resistance emerged in either treatment for Pseudomonas and 100% of *Variovorax* colonies were phage-resistant in every treatment replicate. However, for *Ochrobactrum*, phage resistance was significantly lower where the plasmid was present ($\bar{x}$ = 0.028, 95% CI = 0.0008 to 0.51) compared to absent ($\bar{x}$ = 0.61, 95% CI = 0.3 to 0.84; ANOVA comparing models with and without plasmid: *F*_1,10_ = 9.53, *P* = 0.011; Tukey’s honestly significant difference [HSD]: *P* = 0.04). Plasmid proportions differed between species (ANOVA comparing models with and without species × phage: $x_{2}^{2}\;$= 5.60, *P* = 0.061; ANOVA comparing models with and without species: $x_{2}^{2}\;$= 2,833, *P* < 0.001). Plasmid densities were significantly higher in *Ochrobactrum* populations ($\bar{x}$= 0.937, 95% CI = 0.832 to 0.978) than in Pseudomonas ($\bar{x}$ = 0.0002, 95% CI = 0.00002 to 0.002; *P* < 0.001) or *Variovorax* populations ($\bar{x}$ = 0.001, 95% CI = 0.0004 to 0.005; *P* < 0.001) and were not significantly different between Pseudomonas and *Variovorax* (*P* = 0.185; Fig. 3b). Plasmid proportions were higher when phages were absent (ANOVA comparing models with and without phage: $x_{1}^{2}\;$= 11.97, *P* < 0.001; Fig. 3b; phage present: $\bar{x}$ = 0.154, 95% CI = 0.036 to 0.471; phage absent: $\bar{x}$ = 0.002, 95% CI = 0.0002 to 0.008; *P* < 0.001).

### Species-specific effects of each antagonist.

**FIG 4:**
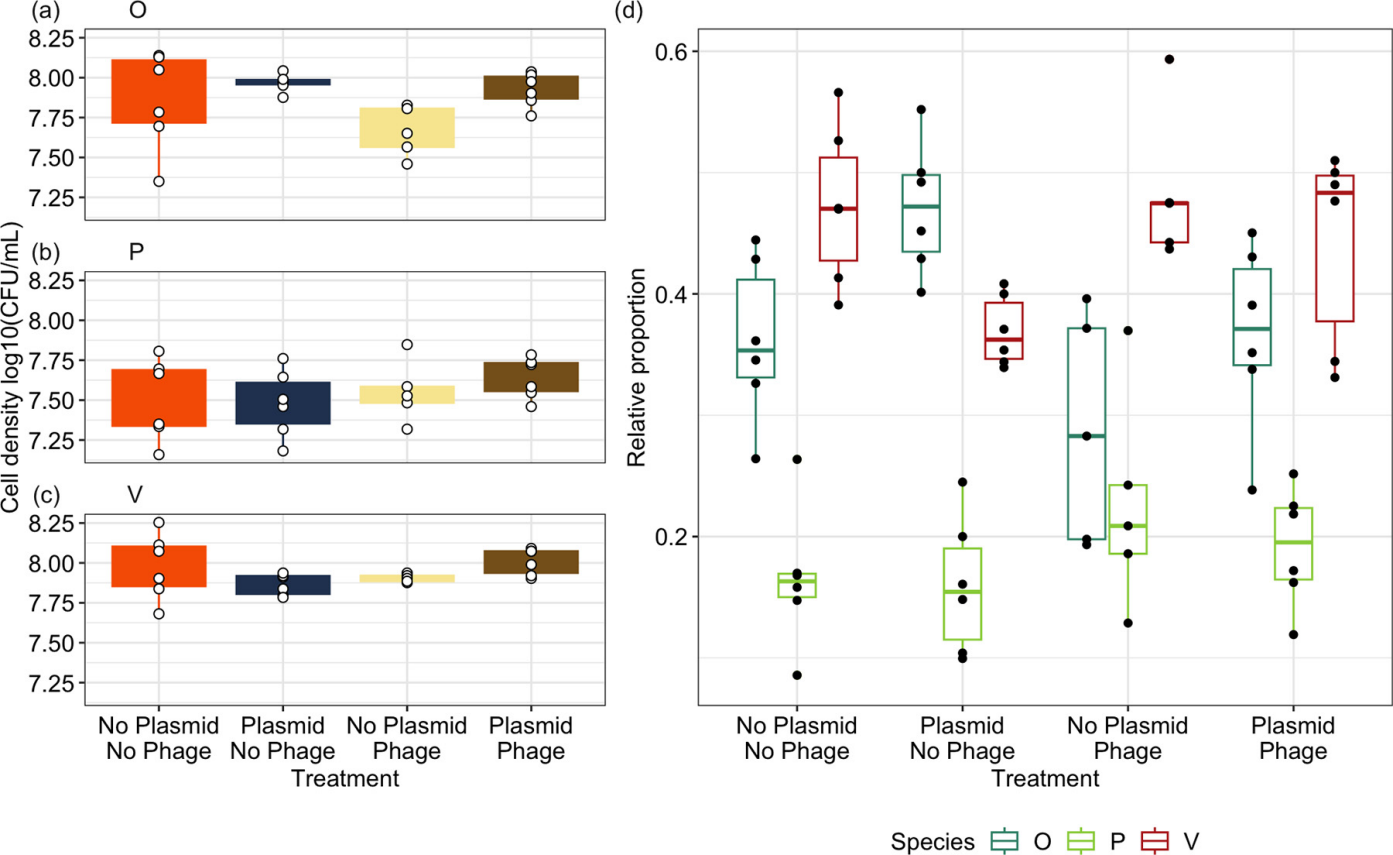
The effects of plasmids and phage on cellular density of (a) *Ochrobactrum* sp. (“O”), (b) Pseudomonas sp. (“P”) and (c) *Variovorax* sp. (“V”) after 2 weeks of culturing. The relative proportion of each species (d) is visualized within each treatment. Points represent individual treatment replicates. Tops and bottoms of the bars represent the 75th and 25th percentiles of the data, white lines indicate the medians, and whiskers extend from their respective hinge to the smallest or largest value no further than 1.5× the interquartile range.

Next, we estimated the effects of single MGEs and pairs of MGEs on bacterial densities within the three-species community. The species-specific effects of each antagonist were evidenced by a significant interaction between species and plasmid presence (ANOVA comparing models with and without species x plasmid: $x_{2}^{2}$ = 7.81, *P* = 0.0201), and between species and phage presence (ANOVA comparing models with and without species × phage: $x_{2}^{2}$ = 9.71, *P* = 0.008). However, these results were almost entirely driven by *Ochrobactrum* density being significantly higher in plasmid-only ($\bar{x}$ = 8.0, 95% CI = 7.88 to 8.13) compared to phage-only communities ($\bar{x}$ = 7.71, 95% CI = 7.57 to 7.84; *P* = 0.033; Fig. 4a). Within species, antagonists did not significantly affect bacterial density between treatment combinations (Tukey’s HSD: *P* > 0.05; Table S2; Fig. 4a to c). The three-way interaction among species, phage, and plasmids (ANOVA comparing models with and without species × plasmid × phage: $x_{2}^{2}$ = 0.59, *P* = 0.746), and the pairwise interaction between phage and plasmids on bacterial density (ANOVA comparing models with and without phage × plasmid: $x_{2}^{2}$ = 2.25, *P* = 0.132) were nonsignificant.

10.1128/mbio.00460-23.4 TABLE S2 Multiple pairwise contrasts comparing species densities within each community combination. The first letter (N, not present; Y, present) indicates phage presence and the second indicates plasmid presence or absence. Different community combinations are separated by an ‘-.’ *P* values adjusted using the Tukey method for comparing a family of three estimates. O, *Ochrobactrum* sp.; P, Pseudomonas sp.; V, *Variovorax* sp. Download Table S2, TIF file, 2.7 MB. Copyright © 2023 Castledine et al. 2023 Castledine et al. https://creativecommons.org/licenses/by/4.0/ This content is distributed under the terms of the Creative Commons Attribution 4.0 International license.

### Effect of antagonists on composition.

Analyzing the relative proportions of each species within communities to account for variation in total densities between replicates revealed a greater impact of antagonists on community composition. Each species was affected differentially by phage and plasmids as indicated by significant pairwise interactions between species and phage presence (ANOVA comparing models with and without species × phage: $x_{2}^{2}\;$= 15.68, *P* < 0.001), and between species and plasmid presence (ANOVA comparing models with and without species × plasmid: $x_{2}^{2}\;$= 14.67, *P* < 0.001). The three-way interaction between species, phage, and plasmid (ANOVA comparing models with and without the three-way interaction: $x_{2}^{2}\;$= 1.46, *P* = 0.482), and the pairwise interaction between phage and plasmid (ANOVA comparing models with and without phage × plasmid: $x_{1}^{2}\;$< 0.001, *P* = 0.980) had nonsignificant effects on species proportions.

The proportion of *Ochrobactrum* was significantly higher in the presence ($\bar{x}$ = 0.416, 95% CI = 0.375 to 0.458) versus the absence of the plasmids ($\bar{x}$ = 0.322, 95% CI = 0.284 to 0.364; Tukey’s HSD: *P* = 0.002), while the opposite effect was evident for *Variovorax* (plasmid presence: $\bar{x}$ = 0.404, 95% CI = 0.364 to 0.446; plasmid absence: $\bar{x}$ = 0.478, 95% CI = 0.433 to 0.523; *P* = 0.018), and plasmids had no significant effect on Pseudomonas (Tukey’s HSD: estimate = 0.111, *z*-ratio = 0.83, *P* = 0.406; Fig. 4d). The presence of phages significantly lowered the proportion of *Ochrobactrum* (phage present: $\bar{x}$ = 0.322, 95% CI = 0.284 to 0.363; phage absent: $\bar{x}$ = 0.417, 95% CI = 0.376 to 0.459; *P* = 0.001) while the proportion of Pseudomonas was significantly higher when phages were present (phage present: $\bar{x}$ = 0.204, 95% CI = 0.175 to 0.236; phage absent: $\bar{x}$ = 0.158, 95% CI = 0.135 to 0.184; *P* = 0.021; Fig. 4d). Phages had no significant effect on the proportion of *Variovorax* (Tukey’s HSD: estimate = −0.187, *z*-ratio = −1.48, *P* = 0.138; Fig. 4d).

While trophic interactions caused changes in species proportions, communities typically followed the same abundance rank orders. In the absence of trophic interactions, the proportions of *Ochrobactrum* ($\bar{x}$ = 0.369, 95% CI = 0.321 to 0.420) and *Variovorax* ($\bar{x}$ = 0.454, 95% CI = 0.403 to 0.507) were not significantly different (Tukey’s HSD: estimate = −0.355, *z*-ratio = −2.313, *P* = 0.054) while both species had significantly higher proportions than Pseudomonas ($\bar{x}$ = 0.166, 95% CI = 0.137 to 0.199; Tukey’s HSD: *P* < 0.001; Fig. 4d). This structure was followed for the plasmid-only and plasmid-phage communities (Table S3; Fig. 4d). However, for phage-only communities, the proportion of *Ochrobactrum* was decreased ($\bar{x}$ = 0.279, 95% CI = 0.236 to 0.328) such that it was not significantly different from Pseudomonas ($\bar{x}$= 0.213, 95% CI = 0.176 to 0.255; Tukey’s HSD: estimate = 0.361, *z*-ratio = 2.137, *P* = 0.0824) with *Variovorax* having a significantly higher proportion ($\bar{x}$ = 0.501, 95% CI = 0.445 to 0.557; Tukey’s HSD: *P* < 0.001) than either species (Fig. 4d).

10.1128/mbio.00460-23.5 TABLE S3 Multiple pairwise comparisons comparing species proportions where plasmids and phage are present (Y) or absent (N). *P* values adjusted using the Tukey method for comparing a family of three estimates. O, *Ochrobactrum* sp.; P, Pseudomonas sp.; V, *Variovorax* sp. Download Table S3, TIF file, 2.4 MB. Copyright © 2023 Castledine et al. 2023 Castledine et al. https://creativecommons.org/licenses/by/4.0/ This content is distributed under the terms of the Creative Commons Attribution 4.0 International license.

### Effects on productivity and evenness.

**FIG 5:**
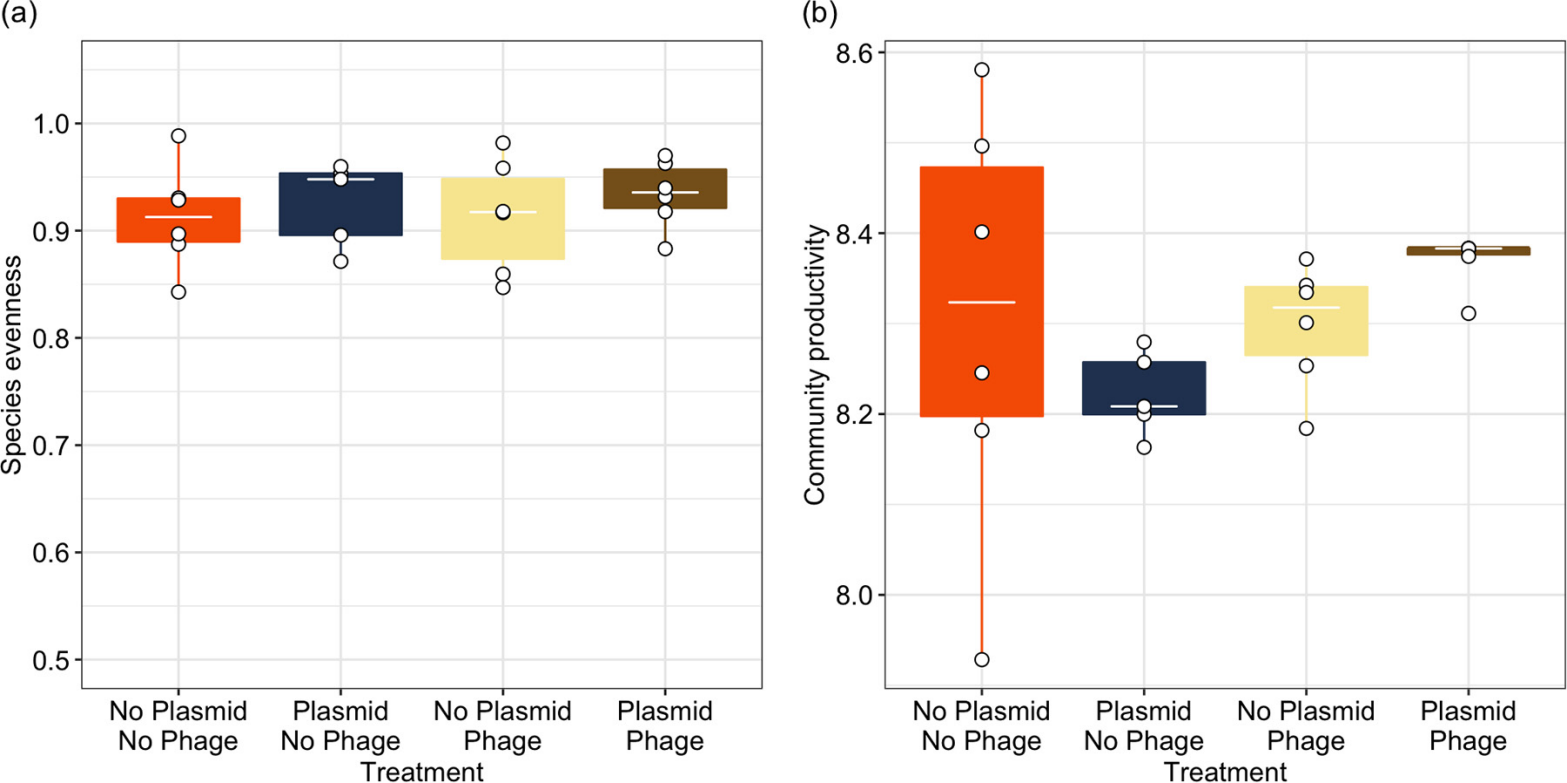
The effect of plasmids and phage on (a) species evenness and (b) community productivity (total community density: log_10_[CFU/mL]) after 2 weeks of culturing. Points represent individual treatment replicates. Tops and bottoms of the bars represent the 75th and 25th percentiles of the data, white lines indicate the medians, and whiskers extend from their respective hinge to the smallest or largest value no further than 1.5× the interquartile range.

Finally, we examined whether trophic interactions had impacts on two community-level properties: species evenness (how equally spread total community density is across the three species) and community productivity (total density of the whole community). Here, there was no significant effect of plasmids (ANOVA comparing models with and without plasmid × phage interaction: *F*_1, 19_ = 0.04, *P* = 0.844; fixed effect of plasmid: *F*_1, 20_ = 0.065, *P* = 0.801) or phage (ANOVA comparing models with and without phage: *F*_1, 21_ = 0.962, *P* = 0.338) on species evenness (Fig. 5a). Similarly, there was no significant effect of plasmids (ANOVA comparing models with and without plasmid × phage interaction: *F*_1, 19_ = 2.069, *P* = 0.167; fixed effect of plasmid: *F*_1, 21_ = 1.495, *P* = 0.235) or phage (ANOVA comparing models with and without phage: *F*_1, 20_ = 0.002, *P* = 0.966) on community productivity (Fig. 5b).

### The effect of phages and the plasmid in monoculture.

**FIG 6:**
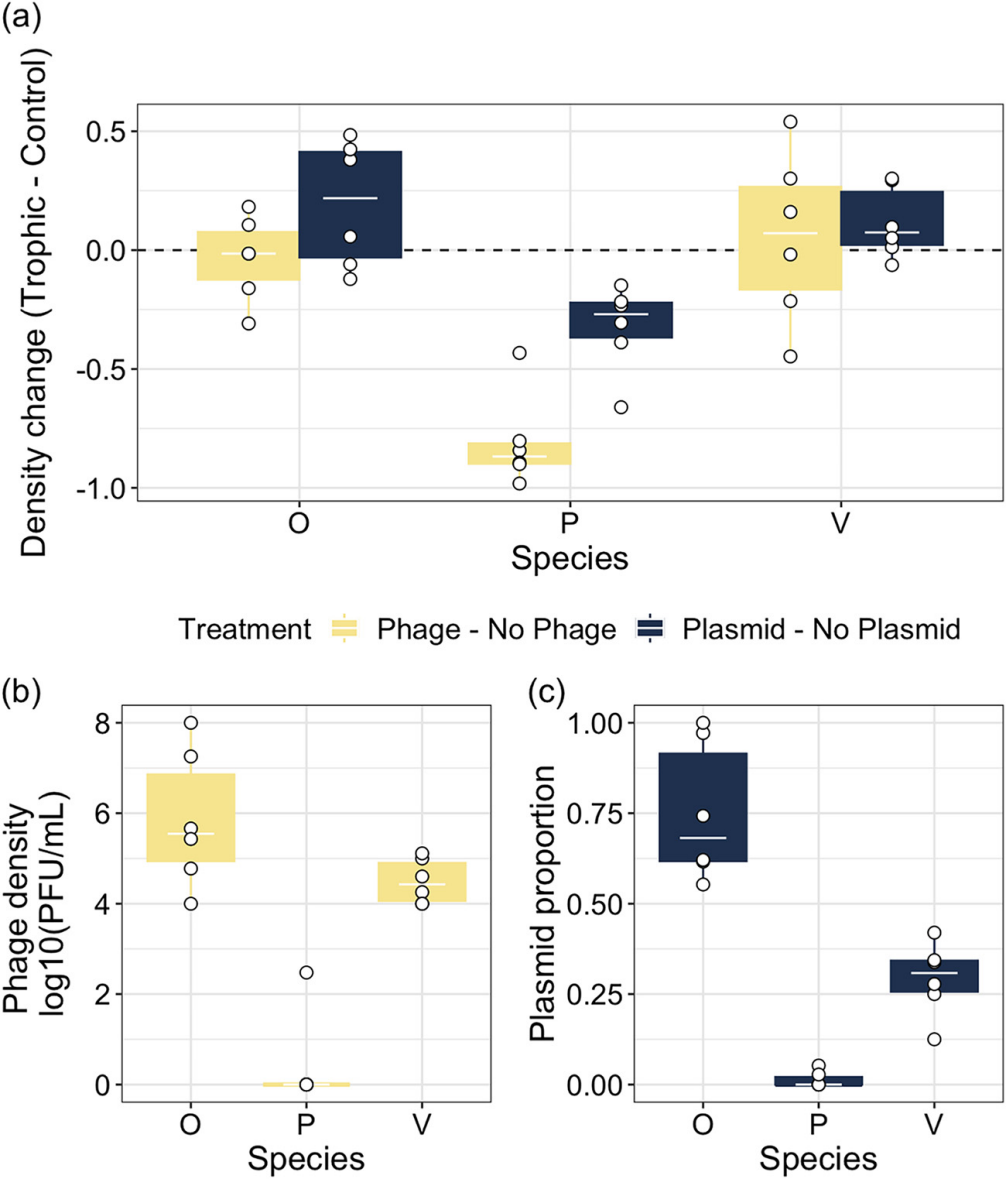
(a) Effects of plasmid and phage presence on species densities in monoculture after 2 weeks of culturing. Dotted line (*y* = 0) indicates where density measured without trophic interactions is equal to the density measured with trophic interactions. A negative value indicates that density is greater without trophic interactions, while a positive value indicates a positive effect of plasmid/phage presence. Points represent individual treatment replicates. The (b) density of phage in each culture and (c) proportion of plasmid carriers are shown. Tops and bottoms of the bars represent the 75th and 25th percentiles of the data, the white lines are the medians, and the whiskers extend from their respective hinge to the smallest or largest value no further than 1.5× the interquartile range. O, *Ochrobactrum* sp.; P, Pseudomonas sp.; V, *Variovorax* sp.

To understand the extent to which the effects of antagonists in polyculture could be driven by bipartite interactions, we measured the effects of phage and plasmids in monoculture (single bacterial species with one antagonist). Neither phage or plasmid had a significant effect on densities for *Ochrobactrum* or *Variovorax* (one-sample *t* tests comparing interaction to 0: *P* > 0.05; Table S4; Fig. 6a). However, the presence of phage (one-sample *t* test: *t* = −10.213, *P* = 0.001) and plasmids (one-sample *t* test: *t* = −4.347, *P* = 0.022) had significant negative effects on Pseudomonas densities compared to that when trophic interactions were absent (Fig. 6a). These effects on Pseudomonas density were observed with phage populations going extinct in all but 1 replicate (Fig. 6b) and complete plasmid loss occurring in 4/6 replicates ($\bar{x}$ = 0.04 in 2/6 replicates; Fig. 6c). These results contrast with our observations in polyculture, where *Ochrobactrum* was affected by both plasmids and phage (in density and proportion) while the proportion of *Variovorax* was affected by plasmids; therefore, these results showed that the effects from antagonists on *Ochrobactrum* and *Variovorax* were dependent on the presence of other species. Additionally, while Pseudomonas was affected by both antagonists in monoculture, it was only affected by phage in polyculture. These effects were not driven by phage resistance, as only 2/6 cultures showed resistance in monoculture at low levels ($\bar{x}$ = 21%, standard error [SE] = 4.2%) and no resistance in polyculture. Additionally, *Variovorax* evolved 100% resistance in all monoculture replicates, similar to polyculture results above while resistance levels for *Ochrobactrum* did not differ between monoculture or polyculture (ANOVA: *F*_1, 9_ = 0.005, *P* = 0.947). Overall, these contrasting results clearly demonstrate a key role of indirect antagonistic and competitive interactions in shaping polyculture composition.

10.1128/mbio.00460-23.6 TABLE S4 One sample *t* tests analyzing differences (trophic versus not trophic) in species density against 0. *P* values corrected for multiple testing using the false-discovery-rate (FDR) method. Lower (LCI) and upper (UCI) 95% confidence intervals are shown. O, *Ochrobactrum* sp.; P, Pseudomonas sp.; V, *Variovorax* sp. Download Table S4, TIF file, 1.2 MB. Copyright © 2023 Castledine et al. 2023 Castledine et al. https://creativecommons.org/licenses/by/4.0/ This content is distributed under the terms of the Creative Commons Attribution 4.0 International license.

## DISCUSSION

Although bacteriophage are highlighted as key drivers of microbial community diversity and function, evidence for this hypothesis is mixed (10). Because phages naturally coexist with other antagonists, we hypothesized that increasing antagonistic MGE complexity would reduce the effect of a given MGE on community composition. Supporting this, our modeling results demonstrate that as the number of antagonists increased, the net effect of antagonists on community composition was reduced. This is because the relative reductions in the density of each species caused by MGEs start to converge. Within experimental communities, each species was differentially affected by phages and plasmids. Plasmids increased the relative proportion of *Ochrobactrum* while decreasing the proportion of *Variovorax*. In contrast, phages decreased the relative proportion of *Ochrobactrum* while increasing the proportion of Pseudomonas. However, when both plasmids and phage were present, the differential effects of each antagonist on *Ochrobactrum* canceled each other out, reverting the community back to its no-trophic structure. The lower levels of phage resistance in the presence of plasmid suggest that the plasmid buffered the effects of phage, thereby reducing selection for resistance. These results suggest that phages can affect community structure, but their effects can be mitigated by the presence of other MGEs and antagonists in general.

The reduced net effect of MGEs when both plasmid and phage were present might also be affected by phages and plasmid directly, or indirectly through competition for hosts, interfering with each other. However, in most cases, neither phage densities nor plasmid prevalence were affected by the other MGE. Plasmids infecting *Ochrobactrum* did, however, decrease in the presence of phage. This may have diminished the positive effect of plasmids in the relative proportion of *Ochrobactrum* within communities, helping drive composition back to an MGE-free state.

The impact of MGEs on host species composition was largely indirect, as apparent from contrasts between the impact of each MGE on bacterial populations in monoculture versus polyculture. For example, the density of Pseudomonas was significantly reduced by phage and plasmid presence in monoculture, yet in polyculture Pseudomonas achieved higher frequencies in the presence of phage. This result is consistent with previous work showing that Pseudomonas is most negatively affected by this plasmid, while *Ochrobactrum* has a higher tolerance (45). As such, neither *Ochrobactrum* or *Variovorax* density were significantly affected by either phage or plasmid presence in monoculture, yet the presence of plasmid or phages in polyculture resulted in reversal of the rank abundances of *Ochrobactrum* and *Variovorax*. In previous work, *Ochrobactrum* was shown to be subject to indirect effects from other bacterial species (46), which seem to be exaggerated in this work by the presence of MGEs. As *Ochrobactrum* decreased in density due to phage, this likely resulted in Pseudomonas benefiting from competitive release (46). Similarly, as *Variovorax* was penalized more by plasmid presence, this may have partially contributed to the relative increase in *Ochrobactrum* (46). This result shows that predictions made from pairwise bacterium-MGE experiments may not predict the effects of a given MGE in polyculture. Such indirect effects are now assumed to be ubiquitous in multispecies communities (47, 48), making it difficult to predict how community complexity will affect pairwise trophic interactions (49, 50).

The fact that composition was affected by plasmids, despite extensive plasmid loss, is suggestive of legacy effects: effects of a species that persist following extinction. Pseudomonas clearly demonstrates legacy effects in monoculture where phage and plasmids are rapidly lost, and yet it is the only species to show a significant density decrease in monoculture. In a previous study, although a virus drove rotifers (predator) to extinction through indirect competition, legacy effects from the predator on algal (prey/host) population size and evolution were evident after its extinction (51). Legacy effects are likely to be accentuated in communities as competition delays population recovery.

Our experimental and simulation results are consistent, where indirect effects of antagonists in interspecies competition likely resulted in antagonistic interactions being canceled out with increasing trophic complexity. However, this pattern would not arise within all communities, as antagonists can interact to enhance deviation from antagonist-free states (52–54). For instance, phage cocktails (multiple phage types present) can result in lower bacterial densities than single-phage preparations (55, 56). Additionally, some MGEs can cooperate, synergistically increasing host fitness and therefore transmission, e.g., resistance to multiple antibiotics can be carried on separate plasmids which together increase fitness under antibiotic therapy (57). Although some MGEs can have positive effects on host density, these effects would still conflict with the presence of neutral and antagonistic MGEs. As such, we believe our results to be generalizable to complex communities containing a mix of interaction types (35). Additionally, species within complex communities are constantly evolving, which can change the strength and direction of interactions (35, 58). We do not expect (co)evolution to significantly alter results because evolution represents a change in interaction which would similarly be canceled out over the expansive network of positive, negative and neutral MGE effects. These changes are especially unlikely to affect community composition when they act on strains within species populations. Phages, for example, typically infect specific strains, and therefore phage resistance is only likely to drive changes in strain abundance within a population and not total population density (3).

The differential effects of antagonists, specifically MGEs, between bacterial species may partially explain why the effects of phages on community composition are variable. Natural communities represent a diverse assemblage of MGEs, each of which has been found to have variable growth rate costs (or benefits) to their hosts (32–35). Most models arguing that phages maintain diversity assume that the costs of phage presence are simply density-dependent (highest costs to the fastest growing/most dense species) (1), which may be an oversimplification. Additionally, studies which have estimated the effects of phages or other MGEs alone may not be representative of dynamics when multiple MGEs are present. Phages may mediate bacterial densities in certain contexts, such as crashes in algal blooms (2, 20) or cholera epidemics (59), but not more broadly. Addressing the role of MGEs in structuring communities using diverse model systems in conjunction with natural systems will be a necessary next step in understanding how multiple antagonists influence community structure in a range of environments. Furthermore, expanding this theory to encompass positive MGEs (e.g., plasmids under antibiotic selection) and temperate phages carrying beneficial genes would determine the broader relevance of our findings.

## MATERIALS AND METHODS

### Simulations.

We simulated the dynamics of multiple antagonists interacting with an otherwise stable community of competing species based on competitive Lotka-Volterra dynamics (60):

(1) $$\frac{dx_{\text{i}}}{\mathrm{dt}}=r_{\text{i}}x_{\text{i}}\left(1-\;\overset{N}{\underset{\text{j}}{\sum }}\alpha_{\text{ij}}x_{\text{j}}\right)$$

where *x* is a vector of species (focal community and antagonists) population densities, *r* is their corresponding intrinsic growth rates, and α is an *N* × *N* matrix of interaction terms. Assuming that the impact of each antagonist on each species in the focal community comes from some distribution (θ) which has finite variance and is constrained to be positive (therefore always having a negative impact in the population dynamics model), it is straightforward to show that as the total number of antagonists increases, the coefficient of variation (CV) of their combined effect on each focal community member (β) tends to diminish. For a community of *n* species with *m* antagonists, the expected mean value of β is E(β) = E(θ)*m*, and the variance is $\sigma_{\text{β}}^{2}\;$= $\sigma_{\text{θ}}^{2}m$. Thus, $\sigma_{\text{β}}$= $\sqrt{\left(\sigma_{\theta }^{2}m\right)}$and CV can be determined by the following equation:

(2) $$\text{CV}\left(X|m\right)=\frac{\surd \sigma_{\theta }^{2}}{\text{E}(\theta )}\frac{\surd m}{m}$$

Therefore, since *m* must be a positive integer and neither E(θ) nor $\sigma_{\theta }^{2}$ depends on *m*, CV(β) must decrease as *m* increases. We investigated *in silico* how increasing *m* affects deviation in community structure (relative abundances at equilibrium) from community structure in the complete absence of antagonists by numerically solving Equation 1 100,000 times (1,000 time steps of Tsitoura’s 5/4 Runge-Kutta method, implemented using DifferentialEquations.jl [61] in the Julia programming environment [62, 63]) for each combination parameter: *n *= 2, 4, (…), 20; *m *= 0, 1, (…), 10; σ = 0.1, 0.1, (…), 1; and *A *= 0.1, 0.2, (…), 1 with *r*_i_ ~ N^+^(0, 1). The interaction matrix was constructed as follows: each focal species interspecific interaction term ~ Uniform (0, *A*/*n*); each individual antagonist impact on a focal species ~ N^+^(0, σ); all effects of focal species on antagonists = 0 (i.e., antagonist population densities are not sensitive to specific focal species); all self-interaction terms and all interactions between antagonists = 1. For each simulation run where *m *> 0, we computed the Bray-Curtis dissimilarity and Kendall rank correlation (measures of the difference in community composition and abundance rank order of species, respectively) between the resulting relative abundances of focal species and the corresponding relative abundances where *m *= 0. Bray-Curtis dissimilarity between two matched vectors of species relative abundances was calculated as 1 to C, where C is the sum over the lower value for each species (64). Kendall rank correlation is given by (*nc* – *nd*)/*n*, where *nc* is the number of concordant (sharing the same rank in a sorted list of relative abundances) species pairs, *nd* is the number of discordant species pairs, and *n* is the total number of species. The results were visualized using Makie.jl (65).

### Bacterial strains, the plasmid, and phages.

Species isolates were originally obtained from soil and identified as *Ochrobactrum* sp., Pseudomonas sp., and *Variovorax* sp. (46). Each species naturally occurs in soil and can stably coexist for several weeks, making this system applicable for studying natural communities (46, 66). Additionally, these species have unique colony morphologies when plated onto King’s medium agar (KB agar) which is ideal for high-throughput experimentation. In addition to wild-type strains, each species was associated with a self-transmissible and broad host range plasmid, the Incp-1ε type plasmid pKJK5::gfp, which confers resistance to tetracycline (67). This plasmid was selected due to its broad-host range, and tetracycline resistance was chosen as a tag because each wild-type strain is tetracycline-susceptible. The generation of plasmid-bearing (TET) strains of each species is outlined in Newbury et al. (45).

Phages were isolated from pig slurry (collected from Gorst Energy Ltd., October 2019). Pig slurry was diluted with demineralized H_2_O at a ~1:1 ratio. Next, 900 μL diluted slurry was extracted and vortexed with 100 μL chloroform (chloroform extraction) (68). Vials were centrifuged at 14,000 × *g* (Progen GenFuge 24D centrifuge) for 5 min and the supernatant was removed. Then, 50 μL of supernatant was added to individual vials of 6 mL KB broth with 100 μL of each bacterium grown separately. Cultures were incubated at 28°C, shaking at 180 rpm for 2 days, and phages were isolated using chloroform extraction. The presence and specificity of phage to each bacterial species was confirmed by spot assays (69). Phage cultures were diluted and 10 μL of each dilution was spotted onto soft agar overlays of each bacterium, with 100 μL of overnight bacterial culture added to 7 mL soft KB agar. Plates were incubated for 2 days, and we confirmed that each phage could only infect one host bacterium. Single-phage plaques were picked and amplified to obtain isogenic phage strains for each species. These are the only phages known to infect these specific bacterial strains.

### Phage genome sequencing and annotation.

Phage were amplified and extracted as described previously from 2-day monocultures (single species host and phage). To increase phage concentration, 7.5 mL of each sample was centrifuged at 10,000 rpm for 5 min; the supernatant was disposed of, and the pelleted sample was transferred to a sterile Eppendorf. The Qiagen DNeasy Ultra Clean DNA Extraction kit reagents and protocol were used to extract gDNA. gDNA concentration was determined using the QuBit dsDNA HS assay kit regents and protocol. Bacterial contamination was checked via PCR using 16S primers 27f 5′-AGAGTTTGATCCTGGCTCAG-3′ and 1492r 5′-GGTTACCTTGTTACGACTT-3′, Thermo Fisher Scientific DreamTaq DNA polymerase, and 0.2 mg/mL bovine serum albumin. Phages were sequenced by MicrobesNG using in-house protocols (70); Illumina sequencers (HiSeq/NovaSeq) were used to create 250-bp paired-end reads, and adapters were trimmed, removing reads with a quality of <15. Reads were adapter-trimmed using Trimmomatic 0.30 with a sliding window quality cutoff of 15 (71) and assessed with FastQC (72) v0.11.9. Each sample was assembled with SPAdes (73) v3.13.0 using the ‘–careful’ flag. The resulting assembly graphs were visualized with Bandage (74) v0.8.1. In all cases, a single high-coverage contig was obtained and extracted from the assembly. Taxonomy was identified using a BLAST search (BLASTn) on the nonredundant sequence database (NCBI). Prokka was used for genome annotation using the PHROG database of viral sequences (75). Where a close match was found (97% sequence identity), the original annotation was used as a reference protein list instead of the PHROG database. Assemblies and data are available from the European Nucleotide Archive (ENA) under accession no. PRJEB55820.

### Phage imaging.

Single-species chloroform extracts of each phage (2 mL) were passed through a 0.22-μm filter to remove bacterial debris. To analyze phages at the ultrastructural level, purified samples were transferred onto pioloform-coated 100-mesh copper EM grids (Agar Scientific, Stansted, United Kingdom) by floating the grids onto droplets containing the phages on parafilm for 3 min. Grids were then washed 3× for 3 min each on droplets of deionized water before contrasting of the adhered structures in 2% aqueous uranyl acetate for 3 min. Excess stain was removed with filter paper and the grids were air-dried before TEM (transmission electron microscopy) imaging. Samples were analyzed using a JEOL JEM-1400 transmission electron microscope operated at 120 kV and images were taken at a nominal magnification of ×40,000 with a digital camera (Gatan Rio16, Ametek, Abingdon, United Kingdom).

### Bacteria, phage and plasmid polycultures.

Isogenic WT and TET bacterial strains were amplified for 2 days in growth medium (1/64 tryptic soy broth [TSB]; diluted with demineralized H_2_O) at 28°C, shaking; TET strains were grown with 50 μg/mL tetracycline to prevent plasmid loss. Cell densities were normalized to 10^5^ CFU/μL (method described by Padfield et al. [46]). Ten μL of diluted culture (10^6^ CFU) of each species was added to vials with fresh growth medium. Four experimental treatments were established: no plasmids or phage (control), plasmids only, phage only, and both plasmids and phage. To each phage treatment, approximately ~10,000 PFU of each phage was added to the relevant vials to achieve a multiplicity of infection (MOI) of 0.01 (to each host bacterium). Cultures were incubated at 28°C, static, with loosened plastic lids. After 7 days, vials were vortexed and 60 μL of culture was transferred to fresh vials (100-fold dilution). The experiment was terminated after an additional 7 days (14 total days, 1 transfer). This community has been previously shown to reach equilibrium within 2 weeks of culturing, with greater variation in densities within the first week (46). Changes in bacterial density between weeks 1 and 2 are presented in Fig. S2. Each week, 900 μL of culture was cryogenically frozen at −80°C with 900 μL 50% glycerol. Phage was extracted at each time point as previously described. To estimate bacterial densities, cultures were plated out from frozen onto KB agar. Where plasmids were present, cultures were additionally plated onto KB with 50 μg/mL tetracycline to estimate plasmid loss rates. Plates were incubated at 28°C for 2 days and colonies counted. Phage densities were estimated from serial dilutions of spot assays as previously described. Phage resistance was determined by picking 12 colonies of each species within each treatment replicate, which were amplified in 150 μL TSB and tested for resistance against ancestral phage via spot assays, as described above.

10.1128/mbio.00460-23.1 FIG S1 Impacts of antagonists on the structure of simulated communities, measured as the Bray-Curtis dissimilarity and Kendall rank correlation between a community with 0 antagonists and one with *m* (*y* axis) antagonists, for a range of typical interspecific (focal-focal) interaction coefficients (*x* axis). Each tile represents the mean value from 100,000 simulations. Shown are results for each combination of parameters: *n* (number of focal species) = 2, 4, (…), 20; m (number of antagonists) = 0, 1, (…), 10; σ (standard deviation of the half-normal distribution from which antagonist impacts are drawn) = 0.1, 0.1, (…), 1; and A (focal species interspecific interaction modifier) = 0.1, 0.2, (…), 1. Download FIG S1, TIF file, 7.6 MB. Copyright © 2023 Castledine et al. 2023 Castledine et al. https://creativecommons.org/licenses/by/4.0/ This content is distributed under the terms of the Creative Commons Attribution 4.0 International license.

10.1128/mbio.00460-23.2 FIG S2 Changes in bacterial density between weeks 1 and 2 of the experiment within each treatment (a to d). Further analyses were conducted on week 2 data to allow the community to reach equilibrium. Tops and bottoms of the bars represent the 75th and 25th percentiles of the data, white lines indicate the medians, and whiskers extend from their respective hinge to the smallest or largest value no further than 1.5× the interquartile range. O, *Ochrobactrum* sp.; P, Pseudomonas sp.; V, *Variovorax* sp. Download FIG S2, TIF file, 5.5 MB. Copyright © 2023 Castledine et al. 2023 Castledine et al. https://creativecommons.org/licenses/by/4.0/ This content is distributed under the terms of the Creative Commons Attribution 4.0 International license.

### Calculations of bacterial diversity.

Species evenness was chosen as the appropriate diversity metric as there were no extinctions (species richness always equaled three). Species evenness is calculated as *J* = *H*/log(*S*), where *H* is the Shannon-Weaver diversity index and *S* is species richness. The Shannon-Weaver diversity index is calculated by the formula $H\;=\;-1\times \left(\mathrm{sum}\left[P_{i}\right]\;\times \;\log\left[P_{i}\right]\right)$, where *P* is the proportion of each species (*i*) within the microbial community (76).

### Bacteria-phage and bacteria-plasmid interactions in monoculture.

To determine the extent to which the effects observed in polyculture (communities) were determined by simple pairwise interactions between bacteria and phage or plasmids, we tested the effect of each antagonist in monoculture (single bacterial species). Bacterial strains (WT and TET) were grown for 2 days and densities normalized as described above. To each WT strain, ~10,000 PFU of phage were added (MOI = 0.01). Control (no antagonists), phage, and plasmid (TET) populations were cultured for 2 weeks as described above. Bacterial, phage, and plasmid densities were calculated as previously described after 2 weeks. Phage resistance was determined as described above.

### Statistical analyses.

All data were analyzed using R v4.0.3 in R Studio (77) and all plots for experimental data were made using the package ‘ggplot2’ (78). Model simplification was conducted using likelihood ratio tests and Tukey’s *post hoc* multiple-comparison tests were used to identify the most parsimonious model using the R package ‘emmeans’ (79).

Phage density (log_10_ PFU/mL) was analyzed against interacting fixed effects of species identity and plasmid presence in linear mixed-effects models. Differences in plasmid proportions were estimated using generalized linear mixed-effects models with a binomial error structure. Because the data sets contained high numbers of ‘0’s and ‘1’s, 0.001 was added and subtracted from values to improve model fit. Plasmid proportion was analyzed against interacting fixed effects of species identity and phage presence. A random effect of treatment replicate was included to account for non-independence in phage and plasmid models.

Bacterial density was analyzed against interacting fixed effects of species identity, phage presence, and plasmid presence with a random effect of treatment replicate (linear mixed-effects model). Differences in community structure were analyzed in generalized linear mixed-effects models with a binomial error structure. Here, species proportion was analyzed against interacting fixed effects of species identity, phage presence, and plasmid presence. Random effects of treatment replicate and an observation-level random effect were included to account for model overdispersion (80). Species evenness and community productivity were analyzed in separate linear models with interacting fixed effects of plasmid and phage presence.

The effects of trophic interactions on species densities in monoculture were analyzed by calculating difference in species densities in the presence versus absence of plasmids or phage. A value of 0 indicates no effect of trophic interactions on species densities. Multiple one-sample *t* tests were conducted to assess whether the density difference was significantly different from 0, and *P* values were corrected for multiple testing using the false-discovery-rate (FDR) method. Phage resistance was analyzed in a general linear model in which the resistant proportion was analyzed against treatment (plasmid versus no-plasmid, monoculture versus polyculture) with a quasibinomial error structure.

### Data accessibility statement.

All data and R codes used in the analysis will be made available on GitHub (https://github.com/mcastledine96/Multiple_MGEs_effects_mBio_2023). Assemblies and sequencing data are available from ENA under accession no. PRJEB55820.
