# Supplementary material for: Antagonistic Mobile Genetic Elements Can Counteract Each Other’s Effects on Microbial Community Composition
Source: mBio. 2023 Apr 6;14(2):e00460-23. doi: 10.1128/mbio.00460-23 (PMC10127636; doi:10.1128/mbio.00460-23)
Supplement: TABLE S1 [file mbio.00460-23-s0003.docx]

| Name | | | Length | | | | Phage | |
| --- | --- | --- | --- | --- | --- | --- | --- | --- |
| 2OG-Fe(II) oxygenase | | | 705 | | | | CHF7MC | |
| anti-repressor Ant | | | 798 | | | | CHF7MC | |
| capsid assembly protein | | | 876 | | | | CHF7MC | |
| deoxynucleoside monophosphate kinase | | | 666 | | | | CHF7MC | |
| DNA ligase | | | 1,065 | | | | CHF7MC | |
| DNA packaging protein B | | | 1,746 | | | | CHF7MC | |
| DNA packaging protein small subunit | | | 258 | | | | CHF7MC | |
| DNA polymerase | | | 2,148 | | | | CHF7MC | |
| endonuclease I | | | 444 | | | | CHF7MC | |
| exonuclease | | | 945 | | | | CHF7MC | |
| Gp5.5-like host HNS inhibition | | | 384 | | | | CHF7MC | |
| head-tail connector protein | | | 1,632 | | | | CHF7MC | |
| internal virion protein A | | | 294 | | | | CHF7MC | |
| internal virion protein B | | | 588 | | | | CHF7MC | |
| internal virion protein C | | | 2,217 | | | | CHF7MC | |
| internal virion protein D | | | 4,179 | | | | CHF7MC | |
| lysozyme | | | 441 | | | | CHF7MC | |
| major capsid protein | | | 1,044 | | | | CHF7MC | |
| nucleotidyltransferase | | | 540 | | | | CHF7MC | |
| primase/helicase protein | | | 1,692 | | | | CHF7MC | |
| putative host RNA-polymerase inhibitor | | | 168 | | | | CHF7MC | |
| RNA polymerase | | | 138 | | | | CHF7MC | |
| RNA polymerase | | | 2,658 | | | | CHF7MC | |
| Rz-like lysis protein | | | 450 | | | | CHF7MC | |
| single-stranded DNA-binding protein | | | 702 | | | | CHF7MC | |
| tail assembly protein | | | 303 | | | | CHF7MC | |
| tail ﬁber protein | | | 357 | | | | CHF7MC | |
| tail ﬁber protein | | | 1,830 | | | | CHF7MC | |
| tail ﬁber protein | | | 2,427 | | | | CHF7MC | |
| tail tubular protein A | | | 588 | | | | CHF7MC | |
| type II holin | | | 216 | | | | CHF7MC | |
| 5'-3' deoxyribonucleotidase | | | 564 | | | | ORM_20 | |
| amidase | | | 882 | | | | ORM_20 | |
| ATP-dependent DNA ligase | | | 1,290 | | | | ORM_20 | |
| baseplate hub | | 702 | | | ORM_20 |  |  |  |
| baseplate hub subunit and tail lysozyme | | 912 | | | ORM_20 |  |  |  |
| baseplate hub subunit and tail lysozyme | | 2,571 | | | ORM_20 |  |  |  |
| baseplate tail tube cap | | 855 | | | ORM_20 |  |  |  |
| baseplate wedge subunit | | 381 | | | ORM_20 |  |  |  |
| baseplate wedge subunit | | 603 | | | ORM_20 |  |  |  |
| baseplate wedge subunit | | 1,482 | | | ORM_20 |  |  |  |
| baseplate wedge subunit | | 1,770 | | | ORM_20 |  |  |  |
| baseplate wedge subunit | | 2,760 | | | ORM_20 |  |  |  |
| clamp loader of DNA polymerase | | 420 | | | ORM_20 |  |  |  |
| clamp loader of DNA polymerase | | 957 | | | ORM_20 |  |  |  |
| dCMP deaminase | | 654 | | | ORM_20 |  |  |  |
| deoxynucleoside monophosphate kinase | | 648 | | | ORM_20 |  |  |  |
| DNA end protector | | 627 | | | ORM_20 |  |  |  |
| DNA helicase | | 1,506 | | | ORM_20 |  |  |  |
| DNA helicase | | 1,719 | | | ORM_20 |  |  |  |
| DNA ligase | | 396 | | | ORM_20 |  |  |  |
| DNA polymerase | | 2,565 | | | ORM_20 |  |  |  |
| DNA polymerase exonuclease subunit | | 834 | | | ORM_20 |  |  |  |
| DNA polymerase processivity factor | | 717 | | | ORM_20 |  |  |  |
| DNA topoisomerase II | | 1,347 | | | ORM_20 |  |  |  |
| DNA topoisomerase II large subunit | | 1,932 | | | ORM_20 |  |  |  |
| DnaB-like replicative helicase | | 1,398 | | | ORM_20 |  |  |  |
| DprA-like DNA recombination-mediator protein | | 483 | | | ORM_20 |  |  |  |
| endonuclease V N-glycosylase UV repair enzyme | | 378 | | | ORM_20 |  |  |  |
| exonuclease | | 702 | | | ORM_20 |  |  |  |
| exonuclease | | 825 | | | ORM_20 |  |  |  |
| head closure Hc2 | | 693 | | | ORM_20 |  |  |  |
| head maturation protease | | 696 | | | ORM_20 |  |  |  |
| head scaffolding protein | | 744 | | | ORM_20 |  |  |  |
| head-tail adaptor Ad2 | | 756 | | | ORM_20 |  |  |  |
| homing endonuclease | | 495 | | | ORM_20 |  |  |  |
| homing endonuclease | | 636 | | | ORM_20 |  |  |  |
| homing endonuclease | | 708 | | | ORM_20 |  |  |  |
| homing endonuclease | | 714 | | | ORM_20 |  |  |  |
| host RecBCD nuclease inhibitor | | | | | 405 | ORM_20 |  |  |
| late sigma transcription factor | | | | | 558 | ORM_20 |  |  |
| lipoprotein | | | | | 840 | ORM_20 |  |  |
| lipoprotein precursor | | | | | 225 | ORM_20 |  |  |
| major head protein | | | | | 1,359 | ORM_20 |  |  |
| membrane protein | | | | | 210 | ORM_20 |  |  |
| metal-dependent phosphohydrolase | | | | | 555 | ORM_20 |  |  |
| MutT/NUDIX hydrolase | | | | | 519 | ORM_20 |  |  |
| nucleotide pyrophosphohydrolase | | | | | 432 | ORM_20 |  |  |
| ParB-like partition protein | | | | | 459 | ORM_20 |  |  |
| portal protein | | | | | 1,659 | ORM_20 |  |  |
| ribonucleoside diphosphate reductase small subunit | | | | | 1,026 | ORM_20 |  |  |
| ribonucleoside-diphosphate reductase large subunit | | | | | 1,827 | ORM_20 |  |  |
| RNaseH | | | | | 909 | ORM_20 |  |  |
| RuvC-like Holliday junction resolvase | | | | | 576 | ORM_20 |  |  |
| Rz-like spanin | | | | | 294 | ORM_20 |  |  |
| SbcC-like subunit of palindrome speciﬁc endonuclease | | | | | 1,638 | ORM_20 |  |  |
| SbcD-like subunit of palindrome speciﬁc endonuclease | | | | | 948 | ORM_20 |  |  |
| single strand DNA binding protein | | | | | 984 | ORM_20 |  |  |
| tail ﬁber protein | | | | | 1,665 | ORM_20 |  |  |
| tail ﬁber protein | | | | | 1,830 | ORM_20 |  |  |
| tail ﬁber protein | | | | | 2,685 | ORM_20 |  |  |
| tail protein | | | | | 1,320 | ORM_20 |  |  |
| tail sheath | | | | | 2,523 | ORM_20 |  |  |
| tail sheath stabilizer | | | | | 846 | ORM_20 |  |  |
| tail tube | | | | | 525 | ORM_20 |  |  |
| tail tube | | | | | 534 | ORM_20 |  |  |
| tail tube | | | | | 624 | ORM_20 |  |  |
| terminase large subunit | | | | | 1,761 | ORM_20 |  |  |
| terminase small subunit | | | | | 492 | ORM_20 |  |  |
| thioredoxin domain protein | | | | | 261 | ORM_20 |  |  |
| thymidylate synthase | | | | | 768 | ORM_20 |  |  |
| thymidylate synthase | | | | | 960 | ORM_20 |  |  |
| tRNA-Asn | | | | | 75 | ORM_20 |  |  |
| tRNA-Ile | | | | | 76 | ORM_20 |  |  |
| tRNA-Met | | | | | 76 | | ORM_20 | |
| tRNA-Thr | | | | | 76 | | ORM_20 | |
| tRNA-Trp | | | | | 74 | | ORM_20 | |
| UvsX-like recombinase | | | | | 1,191 | | ORM_20 | |
| UvsY-like recombination mediator | | | | | 465 | | ORM_20 | |
| virion structural protein | | | | | 219 | | ORM_20 | |
| ATP-dependent DNA ligase | | | | | 861 | | VAC_51 | |
| DNA polymerase | | | | | 2,514 | | VAC_51 | |
| DNA polymerase exonuclease subunit | | | | | 810 | | VAC_51 | |
| DNA primase | | | | | 828 | | VAC_51 | |
| DnaB-like replicative helicase | | | | | 1,275 | | VAC_51 | |
| endolysin | | | | | 510 | | VAC_51 | |
| endonuclease VII | | | | | 435 | | VAC_51 | |
| head scaffolding protein | | | | | 954 | | VAC_51 | |
| head-tail adaptor | | | | | 1,572 | | VAC_51 | |
| HNH endonuclease | | | | | 441 | | VAC_51 | |
| holin | | | | | 261 | | VAC_51 | |
| internal virion lysozyme motif protein | | | | | 2,751 | | VAC_51 | |
| internal virion protein | | | | | 639 | | VAC_51 | |
| internal virion protein with endolysin domain protein | | | | | 3,876 | | VAC_51 | |
| major head protein | | | | | 1,023 | | VAC_51 | |
| nucleotide kinase | | | | | 417 | | VAC_51 | |
| nucleotidyltransferase | | | | | 558 | | VAC_51 | |
| RNA polymerase | | | | | 2,430 | | VAC_51 | |
| RNaseH | | | | | 909 | | VAC_51 | |
| Rz-like spanin | | | | | 204 | | VAC_51 | |
| Rz-like spanin | | | | | 360 | | VAC_51 | |
| short tail ﬁber protein | | | | | 510 | | VAC_51 | |
| tail protein | | | | | 561 | | VAC_51 | |
| tail protein | | | | | 2,361 | | VAC_51 | |
| terminase large subunit | | | | | 1,806 | VAC_51 |  |  |
| terminase small subunit | | 282 | | | VAC_51 |  |  |  |
| virion structural protein | | 291 | | | VAC_51 |  |  |  |
|  | | |  | | | |  | |
