# Supplementary figures and images for: Antagonistic Mobile Genetic Elements Can Counteract Each Other’s Effects on Microbial Community Composition
Source: mBio. 2023 Apr 6;14(2):e00460-23. doi: 10.1128/mbio.00460-23 (PMC10127636; doi:10.1128/mbio.00460-23)

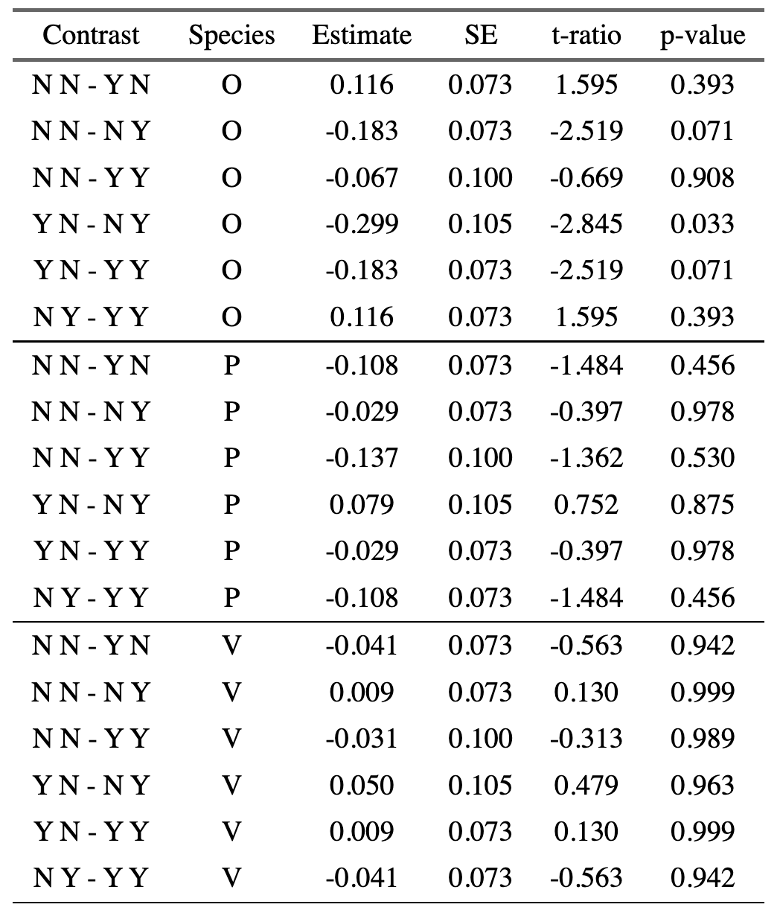

Supplement: TABLE S2 [file mbio.00460-23-s0004.tif]

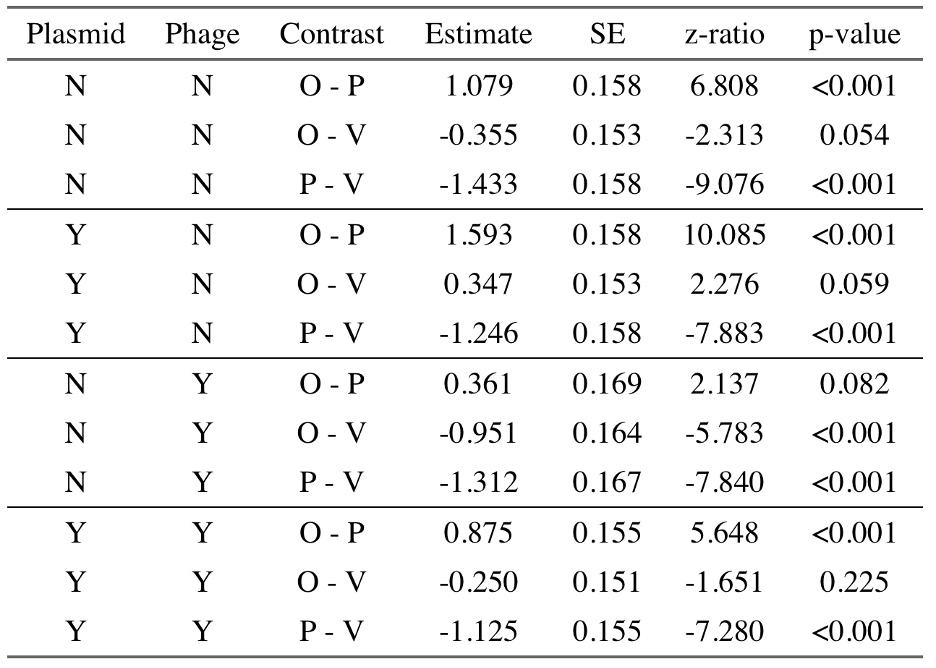

Supplement: TABLE S3 [file mbio.00460-23-s0005.tif]

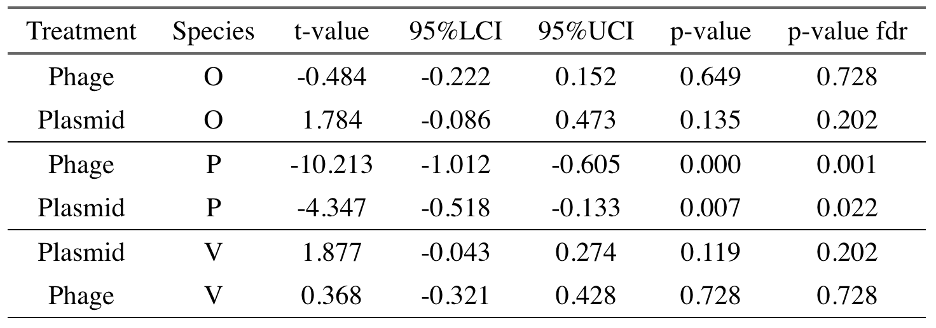

Supplement: TABLE S4 [file mbio.00460-23-s0006.tif]

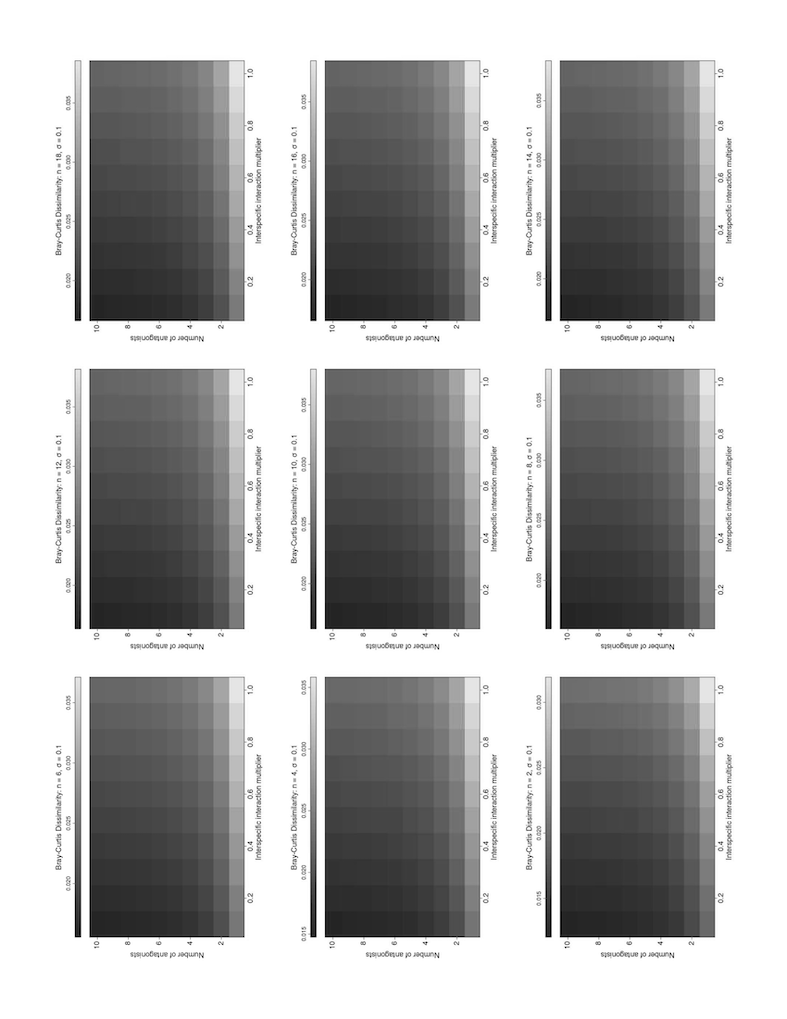

Supplement: FIG S1 [file mbio.00460-23-s0001.tif]

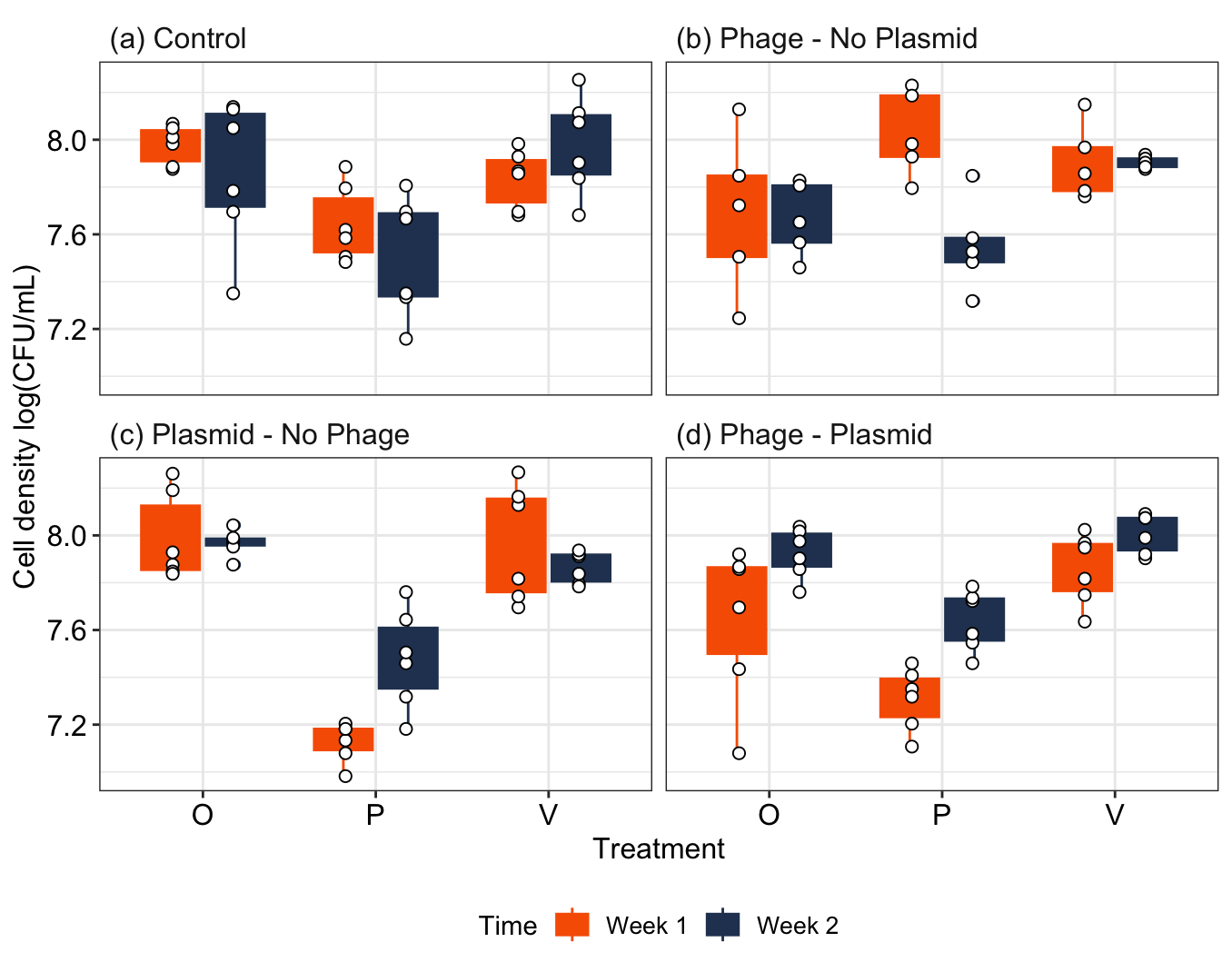

Supplement: FIG S2 [file mbio.00460-23-s0002.tif]
